# Machine Learning based Classification of Ocular Pathogens using Host-transcriptomic Signatures: a proof-of-concept

**DOI:** 10.21203/rs.3.rs-11233042/v1

**Published:** 2026-10-27

**Authors:** Maria Taher Plumber, Ratnesh Kumar, Tejabhiram Yadavalli

**Affiliations:** University of Illinois Chicago; University of Illinois Chicago; University of Illinois Chicago

## Abstract

**Background.:**

Diagnosis of infectious keratitis, uveitis, and endophthalmitis is constrained by the scarcity of pathogen-derived material in small-volume ocular specimens. The host transcriptome is an abundant alternative signal: infection elicits pathogen-class-specific transcriptional responses that may permit pathogen identification without direct detection. We tested this premise as a proof of concept using publicly available in-vitro infection RNA seq. We assembled 330 human RNA-seq samples (177 infected, 153 mock) spanning six pathogens (Herpes Simplex Virus-1, Human Cytomegalovirus Virus, Aspergillus Fumigatus, Candida Albicans, Pseudomonas Aeruginosa, Mycobacterium Tuberculosis) across 36 BioProjects and 24 host cell types.

**Results.:**

Per-study differential expression yielded a robust-gene feature matrix. Pathogen classification was evaluated with Random Forest under leave-one-study-out (grouped) cross-validation and contrasted against random cross-validation to quantify study-level information leakage. Under random (non-grouped) cross-validation, balanced accuracy reached 0.85–0.92 across gene panel sizes (8–700 + genes). Under honest, leakage-corrected leave-one-group-out validation, balanced accuracy was lower than random cross-validation and remained stable across panel sizes and gene-selection strategies, peaking at 0.70 (60–100 gene panel) and averaging 0.519–0.60 across the full range of gene sets tested (8 to 9,942 genes; chance level = 0.14 for 7 classes). Unsupervised UMAP visualization of the robust gene set showed distinct clustering by pathogen and by pathogen class (viral, fungal, bacterial), with cross-kingdom pathogen pairs showing more distinct separation than within-kingdom comparisons.

**Conclusions.:**

Beyond established diagnostics such as PCR and metagenomics, host transcriptomic signatures analyzed via machine learning offer a promising alternative approach to pathogen classification, achieving balanced accuracy substantially above chance under rigorous, study-independent validation. These findings support the feasibility of host-transcriptome-based diagnostic classification, with dataset provenance as the primary current limitation to clinical translation.

## 1. Background

Corneal opacification from infectious keratitis accounts for a substantial share of global blindness, and uveitis, conjunctivitis, and endophthalmitis each carry independent risk of irreversible vision loss (Ting et al., 2021). While herpesviruses carry a disproportionate share of this burden (Farooq and Shukla, 2012), various bacteria and fungi also cause irreversible vision loss if left undiagnosed and untreated. Aspergillus species; including Aspergillus fumigatus (AF) rank among the leading causes of fungal keratitis and endophthalmitis, particularly following ocular trauma or in immunocompromised patients, and is associated with poor visual outcomes when treatment is delayed (Hoffman et al., 2021). Candida albicans (CA) is the most common cause of endogenous fungal endophthalmitis, typically arising from hematogenous spread in patients with candidemia, and requires prompt antifungal therapy distinct from bacterial or viral regimens (Singh et al., 2022). Pseudomonas aeruginosa (PA) causes an aggressive, rapidly progressive bacterial keratitis, especially among contact lens wearers, capable of causing corneal perforation within days if not treated with pathogen-appropriate antibiotics (Dave et al., 2020). Mycobacterium tuberculosis (MTB) causes chronic, frequently misdiagnosed intraocular inflammation that mimics other uveitic and infectious entities, and corneal involvement that is largely hypersensitivity-mediated, making definitive identification essential to avoid prolonged, ineffective therapy (Agrawal et al., 2021). Because the causative pathogen dictates therapy, accurate organism-level identification is essential: empirical treatment directed at the wrong pathogen class not only delays effective therapy but can actively accelerate tissue damage, producing outcomes that are difficult to reverse (Ung et al., 2019). Existing diagnostics each carry constraints. Culture remains the reference standard for bacteria and fungi (Ung et al., 2019) but is slow and positive in only about half of clinically diagnosed cases, and it is impractical for viruses. Direct microscopy (Ting et al., 2022) is rapid but operator-dependent; and PCR is fast but target-specific, requiring the clinician to anticipate the organism, with incomplete primer panels producing false negatives (Taravati et al. 2013). All three diagnostic strategies require pathogen-derived material at detectable levels which may frequently be unmet in aqueous or vitreous volumes of 0.01–0.3 mL. Metagenomic next-generation sequencing (mNGS) is a recent strategy that is gaining traction, whereby the ocular sample is sent to NGS and through a bioinformatic pipeline, the class, species and even antibiotic resistance can be mapped simultaneously (Pardo et al., 2025). However, this strategy also suffers from the scarcity of pathogen derived genomic material especially (during early-stage and ocular infection).

On the other hand, we hypothesize that the human host transcriptome offers an orthogonal signal that can be detected and may help in diagnosing the pathogen. Infections trigger differential transcriptional programs that vary by pathogen class. Examples include interferon-stimulated cascades for herpesviruses (O’Connor and Sen, 2021), C-type-lectin/Dectin programs for fungi (Taylor et al., 2006), and distinct inflammatory signatures for gramnegative bacteria (Ciesielska et al., 2020) which are encoded in host mRNA that is abundant independent of pathogen load. A specimen containing too few fungal reads to register by mNGS still carries a host transcriptome shaped by that infection. If the host response is sufficiently pathogen-specific, the organism can be inferred from the host rather than detected directly. In this study we test this using robust genes, those whose direction of change is reproducible across independent studies of the same pathogen and machine learning classifiers trained on them, offering a third route alongside pathogen-detection methods. The samples analyzed here are not clinical ocular specimens, but heterogeneous in-vitro infections taken from openly available Sequence Read Archive (SRA) datasets in National Center for Biotechnology Information (NCBI). The pathogen classes separate even under this constraint indicates conserved host biology rather than chance, and matched ocular tissue should sharpen the signal further.

## 2. Methods

### 2.1 Data assembly and preprocessing

Raw reads were retrieved from NCBI Sequence Read Archive by accession (Leinonen et al., 2010), organized by SRR and BioSample, and downloaded with fasterq-dump from the SRA Toolkit (NCBI, n.d.). Adapters and low-quality bases were trimmed with fastp (Chen et al., 2018). Trimmed reads were pseudoaligned to the human reference transcriptome with kallisto, producing per-sample transcript-level abundances (Bray et al., 2016). Where multiple runs belonged to one BioSample, abundance files were merged so each biological replicate is represented once.

### 2.2 First Feature Matrix and Visualization

Per-sample transcript-level abundances were aggregated to gene level by summation across isoforms. Transcripts absent from the annotation were excluded, retaining 82.2% of quantified transcripts and 35,300 genes. They were assembled into a single matrix with samples as rows and genes as columns, spanning all pathogens and mock controls across every included study. Counts were normalized to transcripts per million to remove differences in library size and transcript length, and log-transformed to stabilize variance across the expression range. Before any feature selection, principal component analysis (PCA) was performed on this full matrix using unfiltered 330 samples × 35,300 gene matrix based on their expression, providing an unsupervised view of the dominant sources of variation in the pooled data. Samples were colored by pathogen to assess whether infection identity was reflected in the leading components prior to any filtering. (Figure 1 a.). The first, colored by pathogen, showed no clear separation between pathogen groups. Infected and mock samples overlap extensively across all pathogens; bacterial samples of PA are broadly dispersed, while viral (HCMV, HSV-1) and fungal (AF, CA) samples along with bacterial samples of MT show minimal separation. This suggested that pathogen identity alone does not explain the dominant axes of variance in the raw data. To investigate this further, we generated a second projection using the identical PCA embedding, this time colored by cell types (Figure 1 b), in order to determine whether samples cluster according to cell types rather than biological condition. Despite 24 independent cell types contributing samples, several cell types form tight, visually distinct clusters (e.g., the green, orange, brown clusters) rather than blending into a single continuous cloud, indicating that study of origin is a meaningful source of variance in raw, unfiltered gene expression data. Some overlap between studies was also present, suggesting that additional factors, such as BioProjects (study types), likely contribute to the observed variance alongside cell type identity. Together, these two projections indicate that in the absence of any correction, raw transcriptomic variance is driven more strongly by technical and experimental factors such as cell type and study type rather than by pathogen-specific biological signal alone.

### 2.3 Differential expression and robust-gene selection

Per-pathogen expression matrices were assembled from infected and mock samples. Differential expressions were computed per BioProject with PyDESeq2 (Muzellec et al., 2023), comparing infected against BioProject-matched mock controls, yielding per-gene log2 fold change (LFC) and adjusted p-values. For each pathogen, genes were evaluated for reproducibility across its constituent studies: a gene was considered significant in a given study if it met standard thresholds for differential expression (padj < 0.05, |log2FC| ≥ 1), and was classified as robust for that pathogen only if it met this threshold with a consistent direction of effect across multiple independent studies. To place the pathogen-associated genes in the context of the broader transcriptome, all genes in the gene-level expression matrix (n = 35,300) were embedded using UMAP. Each gene was represented by a six-dimensional vector of its average log2 fold-change in each pathogen relative to mock. Each point represents one gene, colored by whether it was exclusively associated with a single pathogen’s robust gene set, shared across two or more pathogens, or filtered out (not robust for any pathogen). The huge portion of genes fall into the filtered-out category, while pathogen-exclusive and shared genes are interspersed throughout the embedding rather than forming distinct, separated regions. These values were calculated as the difference in mean log2(TPM+1) between a pathogen’s infected and mock samples, pooled across all contributing studies. UMAP (n_neighbors = 30, min_dist = 0.3) was then applied to these vectors, yielding a two-dimensional embedding in which genes with similar cross-pathogen response patterns lie close together (Figure 1 c).

Genes were categorized separately by their membership in the per-pathogen robust gene sets, which were derived from per-study PyDESeq2 analysis (padj < 0.05, |log2 fold-change| > 1), reproducible across at least two independent studies per pathogen). Genes belonging to a single pathogen’s robust set were labeled pathogen-exclusive, those belonging to two or more were labeled shared, and the remainder were labeled filtered-out. Embedding coordinates were therefore determined independently of robust gene status, which was used only to color points in the visualization.

As the required number of studies increased, fewer genes met this criterion, since consistent effects become harder to sustain across a larger and more diverse set of datasets. These robust genes, selected at the maximum reproducible threshold for each pathogen, were then carried forward as the feature set used for downstream pathogen classification.

For HSV-1, all six available studies were required, yielding 151 robust genes a comparatively high number consistent with the larger overall pool of differentially expressed genes observed for this pathogen. For HCMV, requiring robustness across all seven studies yielded only 38 genes, and separation using this smaller set was poor; requiring robustness across six of seven studies instead yielded 292 genes and produced substantially better separation, and this threshold was therefore used. For AF, five of six studies gave the best separation and was selected accordingly. For CA, four of seven studies yielded 47 robust genes; increasing the threshold to five studies reduced this to only 17 genes, which was judged too small and imbalanced relative to the other pathogens, so four studies was retained. For MT, six of seven studies yielded a sufficient and consistent gene set. For PA, robust genes were only recoverable at a threshold of three of five studies; requiring four or five studies returned zero robust genes, making three studies the only viable threshold for this pathogen.

During any sequencing process, so-called ‘batch’ effects can arise from differences in laboratory conditions, reagent lots and personnel, and become a substantial source of error when correlated with the outcome of interest (Leek et al., 2010; Davies et al., 2021). These batch effects can generate biases when they are collinear with the biological covariates of interest, such as the anatomical location from which the sample was taken, sex or age. In disease studies in particular, it is challenging to avoid collinearity of batch effects and disease conditions. Removing this technical effect during preprocessing may, however, also remove the biological signal, thus affecting the conclusions that can be drawn from such studies. This challenge, the risk that explicit batch correction may remove genuine biological signal along with technical noise, particularly when batch is collinear with disease condition has been noted primarily in single-cell contexts (Willem et al., 2025), but we considered the same concern relevant to our bulk RNA-seq setting. Rather than applying explicit batch correction using cell type and study as covariates, we instead relied on cross-study reproducibility as an implicit filter, reasoning that this approach would better preserve genuine, generalizable biological signal: genes that failed to remain robust across multiple independent studies were treated as more likely to reflect study or batch specific effects than true infection response.

We then generated an UpSet plot (Lex et al., 2014) to visualize the differentially expressed genes (DEGs) across pathogens, identifying how many genes were exclusive to each pathogen versus shared across combinations of two, three, four, five, or six pathogens. For this visualization, genes were required to be robust across minimum number of studies per pathogen (Table 1), a threshold chosen to illustrate the overall scale and distribution of candidate genes across pathogens (Figure 1 d). Vertical bars (top) show the number of DEGs shared across each specific combination of pathogens, indicated by the connected dots below each bar; a single filled dot denotes genes exclusive to that pathogen, while connected dots denote genes shared across the corresponding pathogen combination based on 651 chosen genes. The largest intersection (n=292) corresponds to genes (exclusive and shared) to HCMV, reflecting its substantially larger total DEG count chosen across studies relative to other pathogens.

To further characterize the scale of these robust gene sets, we plotted the total number of robust genes per pathogen at this threshold, divided into genes exclusive to a single pathogen versus genes shared with one or more other pathogens (Figure 1 e.). This provides a clearer view of how genes are distributed at the two-study threshold, and of the overall magnitude of candidate genes obtained before applying stricter, pathogen-specific reproducibility requirement

Infected and mock samples were then compared within each pathogen using that pathogen’s own robust gene set, isolating the infection response from the cell-type and study background common to both groups. Principal component analysis on these restricted feature sets separated infected from mock samples along the leading component for every pathogen, with ellipses drawn at 1.5 standard deviations per group. (Figure 2 a). The degree of separation varied by pathogen, with some showing complete resolution of the two groups and others retaining partial overlap at the boundary, but in all six cases the dominant axis of variation corresponded to infection status rather than to residual structure within the mock samples. The separation of samples in individual pathogen, before robust gene selection, based on the entire gene panel, giving 6 plots total are in Supplementary Data : Additional Figure A1.

### 2.4 Second Feature Matrix and visualization

Robust genes across all six pathogens were combined into a unified matrix in which each row is a sample (infected or mock) and each column a robust gene, the value being that sample’s log fold change relative to its own study’s mock mean. The resulting gene-level values were log_2_-transformed after addition of a pseudocount of 1 (log_2_TPM = log_2_[TPM + 1]), the pseudocount being applied so that genes with zero expression remain defined. Within each study, a reference profile was computed as the mean log2 expression of that study’s mock samples, and each infected sample was expressed as its deviation from that reference. For mock samples, the reference is the mean of all mock samples within the same study, including the sample itself; consequently mock log fold changes are centered near zero by construction. This baseline was intended to place each infected sample in the context of uninfected cells from the same study, allowing infection responses to be compared across pathogens; mock-sample performance is not read as an independent estimate. The analyses below uses 330 samples × 605 unique robust genes for supervised and unsupervised machine learning techniques (Table 1). Robust gene sets from the six pathogens comprised 651 genes in total, with a number of genes repeated for more than one pathogen. As the matrix contains log fold changes for every sample across all pathogens, duplicate entries were removed and the union of the six sets was retained resulting in 605 genes.

To visualize how pathogens separate in the robust-gene space, Uniform manifold approximation and projections (UMAP) (McInnes et al., 2018) were generated by incrementally adding filtered samples from each pathogen, one at a time, alongside the pooled mock samples based on 605 robust genes. Each step was embedded independently, so coordinates are not comparable across panels. (Figure 2 b).

Separation of infected from mock samples after restriction to robust genes confirmed that the selection criterion recovers genes capable of distinguishing infection status, the objective of this step. Robust genes from all six pathogens were then pooled and the full 330 × 605 matrix projected by UMAP. Given the heterogeneity of the assembled data: differences in cell type, sequencing platform, time post-infection, and other technical factors, a substantial overlap between pathogens was anticipated even after filtering. Instead, the pathogen groups resolved into largely distinct clusters, with limited overlap between infected samples and mocks and between pathogens. The same projection was examined by pathogen class and by cell type. Grouping by class was likewise well resolved, while cell types were distributed across rather than within pathogen clusters, indicating that the observed structure reflects the host response to infection rather than the cell-type composition of the underlying studies (Figure 3).

Up to this point, the entire analysis was performed using unsupervised machine learning, relying on established Python and R tools to assess whether pathogens could be separated using differential gene expression alone. The biological plausibility of this separation, together with validation against known marker genes, gave us confidence that the underlying approach was sound, even though the data carried substantial bias. We next turned to supervised machine learning approach to evaluate what additional separation it could provide.

### 2.5 Classification and validation

Pathogen identity (six classes, optionally with a pooled Mock class) was predicted with Random Forest (RF) (Breiman, 2001) and linear Support Vector Machine (SVM) (Cortes & Vapnik, 1995). The primary metric is balanced accuracy under leave-one-group-out cross-validation (LOGO): all samples from a held-out BioProject form the test fold, so no study is split across train and test. When feature selection was applied (top-N genes by RF importance), it was performed strictly within each training fold. As a leakage diagnostic, we additionally report standard random (non-grouped) cross-validation; the gap between the two quantifies how much apparent performance depends on seeing other samples from the same study.

## 3. Results

### 3.1 The cohort spans six pathogens across many studies and cell types

The assembled cohort comprises 330 samples (177 infected, 153 mock) across six pathogens, 36 BioProjects, and 24 host cell types (Table 2). This breadth is the cohort’s strength for generalization testing and, as shown below, the source of its central confound: each pathogen is studied predominantly in a distinct set of host cell types.

### 3.2 Honest cross-study validation vs Random split.

To assess whether the robust gene panel contributed beyond generic feature selection, classification was first performed on the full gene space (35,300 genes) without restriction to the 605-gene panel. At each panel size, the top-N genes were selected by random forest importance within each training fold, so that held-out studies did not contribute to gene selection. Under leave-one-group-out (LOGO) validation, balanced accuracy ranged from 0.519 to 0.586 across panels of 8 to 605 genes, compared with 0.756 to 0.872 under random cross-validation (Table 3). Since the results for the full gene space came much above chance, we decided to use our robust gene panel across studies. Restricting selection to the 605-gene robust panel increased LOGO balanced accuracy to a maximum of 0.709 at 60 genes (Table 4). Because the robust panel was derived using all studies, this difference represents an upper bound on the panel’s contribution.

Using all 605 genes, balanced accuracy under grouped five-fold validation was 0.628(RF) and 0.779 (SVM) for 605 genes for the seven-class problem, far below the 0.90+ obtained under random cross-validation. Both values remain well above the chance level of 0.143 (1/7), indicating that genuine, study-independent pathogen signal is present in the host transcriptome and is not merely an artifact of batch structure (Figure 4 a). However, the substantial gap between honest and leaky evaluation demonstrates that a large share of the apparent classification performance reported under leaky evaluation reflects study-specific confounding rather than true biological discriminability. This pattern is consistent with cross-study assessments in genomic prediction more broadly, where even correctly performed within-study cross-validation yields optimistic accuracy relative to validation on independent datasets (Bernau et al., 2014); the magnitude of the gap observed here, however, is considerably larger than the ~0.06 shift reported in that setting, reflecting the strong collinearity between study and pathogen identity in publicly assembled corpora. Comparable bias has been reported in simulation studies of classifier evaluation, where cross-validation performed on data combining multiple batches yielded performance estimates substantially higher than those obtained on independent data, with the magnitude of the bias increasing as batch and class membership became more confounded ((Soneson et al., 2014).

Per-class recall at a 60-gene panel (Table 5) localizes where the classifier succeeds and fails. The pooled Mock class (0.99) came almost perfectly across held-out studies, and five of the six pathogens: Aspergillus (0.89), HCMV (0.81), and HSV-1 (0.82) generalize reasonably well, Candida (0.61) and Mycobacterium (0.64) are intermediate, showing partial but incomplete transfer. Pseudomonas (0.21), by contrast, does not generalize at all under LOGO validation, indicating that whatever signal distinguishes it within a given study fails to hold across independent datasets (Figure 4 b). Taken together, these results show that “infected versus not” is reliably recoverable, and that a genuine, study-independent pathogen-specific signature exists for most of the six pathogens but this signature is effectively absent for Pseudomonas, the pathogen with the fewest contributing studies (five) in the dataset.

Our worst performing class was Pseudomonas Aeruginosa and then Candida Albicans. For Candida Albicans, 39% was going to mock where mocks were compared against themselves which made all the mock values centered around zero since the major goal was to get the separation between infections compared to mocks. So to see the pathogen separation only, we checked the classification for infected samples and they came up pretty well. We again plotted the umap with just infected sample separation and the clusters were still forming with just a little overlap which is provided in Supplementary Data : Additional Figure A2. This proved the mock dominance in the classifier.

### 3.3 Cross-pathogen summary of selected genes and heatmap visualization

Feature selection was performed with a random forest classifier under grouped five-fold, and the genes selected in each fold were recorded. Genes selected across folds were retained, giving 142 genes, and summarized as a pathogen-by-gene matrix displayed as a dot plot (Figure 4 c). For each pathogen, samples of that pathogen were grouped, and the mean log_2_ fold change was computed per gene across all samples of the group, without filtering, and encoded as cell color on a diverging scale bounded at the 99th percentile of absolute mean fold change. Mock samples were excluded. The proportion of samples within each group reaching an absolute log_2_ fold change of 1 was computed in parallel and encoded as circle diameter, circles being drawn where this proportion reached 0.15 (15% of samples). Genes were ordered by average-linkage hierarchical clustering on Euclidean distances between their cross-pathogen response profiles.

### 3.4 Projection of representative pathogen-specific and shared genes onto the sample-level embedding.

For each of the 142 genes selected across cross-validation folds, per-sample absolute log_2_ fold change was overlaid on the fixed sample-level UMAP embedding (330 samples, including mock). Samples with |log_2_ fold change| ≥ 1 were colored on a red scale proportional to magnitude, capped at 4, and samples below this threshold were shown in blue (Figure 5 a). Representative genes were then selected by visual inspection of these overlays: for each pathogen, a gene whose responding samples were concentrated in that pathogen’s cluster, and for each pathogen class, a gene responding in both member pathogens. Genes were arranged by pathogen class (virus, fungus, bacteria), with pathogen-specific genes in the outer columns and shared genes within the kingdom in the center column. The expression of all 142 genes in different pathogen samples are in Supplementary Data : Additional Figure A3.

### 3.5 Selective sampling and feature engineering might rescue generalization

To examine how sample composition affects performance, classification was repeated on subsets retaining the samples with the largest overall response magnitude (Euclidean norm of the log_2_ fold-change vector) within each class (Table 6). Analyses used all 330 samples across seven classes, with the top 60 genes selected by random forest importance within each training fold, and were evaluated by grouped five-fold and random cross-validation. With all samples, balanced accuracy was 0.633 under grouped cross-validation and 0.904 under random cross-validation. Retaining the top 50 samples per class gave 0.692 and 0.898, and retaining the top 20 per class gave 0.786 and 0.929 (Figure 5 b). Because the Mock class (153 samples) was reduced to 50 and then 20 samples, whereas all pathogen classes (25–35 samples) were fully retained at the top-50 level, so both class balance and response strength contribute to this gain.

Across our various analyses, different gene panel sizes, classifiers, and sample subsets, honest LOGO balanced accuracy with RF for the full six-pathogen, all-samples condition consistently falls within a narrow band of approximately 0.63 to 0.70, never exceeding this upper bound nor dropping meaningfully below it. This stability is itself informative: it indicates that the recovered signal reflects a real, reproducible biological ceiling rather than an artifact of any single modeling choice. Critically, this range sits well above the chance level of 0.143 (1/7 for the seven-class problem), demonstrating that host transcriptomic profiles retain genuine, study-independent information about pathogen identity, and that machine learning classifiers can recover this biological signal even under the most conservative, leakage-free validation scheme available.

## 4. Discussion

This study demonstrates that host transcriptomic signatures carry genuine, reproducible information about ocular pathogen identity. Under LOGO validation, the most conservative evaluation available, since it requires the classifier to generalize to studies it has never seen, balanced accuracy for the six-pathogen task consistently reached 0.63 to 0.70 across gene panel sizes and classifiers, well above the 0.143 chance level for a seven-class problem. Five of the six pathogens (Aspergillus, HCMV, HSV-1, and to a moderate extent, Mycobacterium and Candida) showed strong, consistent per-class recall across held-out studies, and unsupervised projections built from cross-study robust genes showed clear, biologically coherent separation compared to unfiltered gene sets, confirming that the reproducibility-based feature selection is capturing real signal rather than noise.

Gene panel size had only a modest effect on supervised classification accuracy: LOGO balanced accuracy varied narrowly between approximately 0.63 and 0.69 across panel sizes ranging from roughly 40 to the full 605-gene set, with the highest supervised value occurring near 60 genes. Because this difference was small and did not track cleanly with panel size, we selected the full 605-gene robust panel for downstream analysis, as it also produced the clearest and most consistent unsupervised separation across all six pathogens in PCA and UMAP projections. This choice reflects a preference for the panel that performed best across both supervised and unsupervised evaluation jointly, rather than the panel that maximized a single metric in isolation.

We also compared this honest performance to accuracy under naive random cross-validation, which reached 0.90 or higher. This is a useful contrast rather than a discouraging one as it shows precisely how much of the apparent separability in this kind of pooled, multi-study data comes from study-specific structure that a model can exploit if allowed to, and it demonstrates why LOGO validation is the right standard for this class of problem. Reporting both numbers together, rather than only the higher one, gives a realistic and defensible estimate of what this approach can deliver on unseen data, rather than an inflated one.

Pseudomonas was the clear outlier. With only five contributing studies compared to six or seven for the other pathogens, cross-study robust gene selection was directly limited by data availability. This required replication across three studies which yielded roughly 40 genes, and stricter thresholds returned essentially none. Classification performance for Pseudomonas was correspondingly weaker than for the other five pathogens. This is best understood as a dataset coverage issue specific to Pseudomonas rather than a limitation of the method itself, since the same approach performed well for every pathogen with adequate study depth.

Selective sampling by transcriptomic response magnitude raised honest accuracy from 0.633 to 0.786, while the random-split estimate stayed near or above 0.90. The subsets reduced the Mock class as well as retaining the strongest responders, so both class balance and response strength contribute to this gain. What it points to is that samples vary widely in how strongly they respond, and that this variation is part of what limits the pooled estimate. A cohort with standardized infection conditions and more evenly represented classes would likely do better than the figure reported here. Notably, these results were obtained entirely from publicly available datasets deposited on NCBI, assembled from independent laboratories, cell types, and sequencing platforms, rather than from a purpose-built, standardized cohort. That meaningful, above-chance, cross-study-generalizable classification is achievable under these comparatively unfavorable conditions suggests that host transcriptomic profiling, refined with a properly curated and expanded dataset, represents a plausible complementary approach to existing diagnostic modalities such as PCR and mNGS. Of these, mNGS is the most versatile. It is hypothesis free, so the clinician does not need to anticipate the organism, and a single run can report class, species, and in principle, antimicrobial-resistance markers together (Street et al., 2022; Cao et al., 2026). Its limitation follows from reading the pathogen itself, whose nucleic acid is scarce in low volumes that are typically acquired from ocular sampling and are even scarcer in early infections. Host-transcriptomic classification works around this bottleneck as the infection-shaped host signal is abundant regardless of the pathogen load, so a specimen carrying too few pathogen reads to be registered by mNGS can still be assigned from the host response. When sequencing is performed at the RNA level, both readouts come from one run. Human reads make up the majority of an ocular mNGS library and are set aside to recover the low pathogen fraction, so the host signal our classifier uses is the material mNGS discards. mNGS in turn has its own interpretive burdens, since detected nucleic acid doesn’t mark active disease, and separating a live pathogen from residual DNA, a colonizer, or reagent contamination, leans on thresholds and controls rather than a read count alone (Delbeke et al., 2021). This work is intended as an early demonstration of feasibility along that path, rather than a finished diagnostic tool.

### Limitations.

Robust gene selection depends on the number of contributing studies per pathogen, which directly limited Pseudomonas and is the most likely explanation for its weaker performance relative to the other five pathogens. Host cell type was not held constant across pathogens, each pathogen was drawn predominantly from the cell type(s) in which it is most commonly studied, so some fraction of apparent pathogen-specificity may reflect cell-lineage identity rather than infection biology alone. The Mock class was also pooled across heterogeneous studies and cell types, which may understate the difficulty of distinguishing infected from uninfected samples in a more standardized setting. Because mock log fold changes were computed against a baseline that includes the sample itself, the present design was intended for discrimination among infected samples, and mock classification performance should not be taken as an independent estimate. A hierarchical classifier that first distinguishes infected from uninfected samples and then assigns the causative pathogen is planned to address this directly. A classifier built on the host response alone also cannot separate an infection from sterile inflammation such as non-infectious uveitis, so it complements rather than replaces direct detection assays for that distinction. Expanding study coverage for underrepresented pathogens, testing the approach on a matched-tissue cohort where host cell type is held constant, and developing a hierarchical classifier that first separates infected from uninfected samples are natural directions for addressing these constraints.

## 5. Conclusion

Beyond established diagnostics such as PCR and metagenomics, host transcriptomic signatures analyzed via machine learning offer a promising alternative approach to pathogen classification, achieving balanced accuracy substantially above chance under rigorous, study-independent validation, even when built entirely from heterogeneous, publicly available data. These findings support the feasibility of host-transcriptome-based diagnostic classification, with expanded study coverage for underrepresented pathogens such as Pseudomonas aeruginosa, and reduced host-cell-type heterogeneity achievable through a dedicated clinical cohort, as the primary paths to improving performance beyond what is reported here.

## Supplementary Material

This is a list of supplementary files associated with this preprint. Click to download.

• SupplementaryData.pdf

• studydesign330.xlsx

• combinedlfcmatrix.tsv

## Figures and Tables

**Figure 1:**
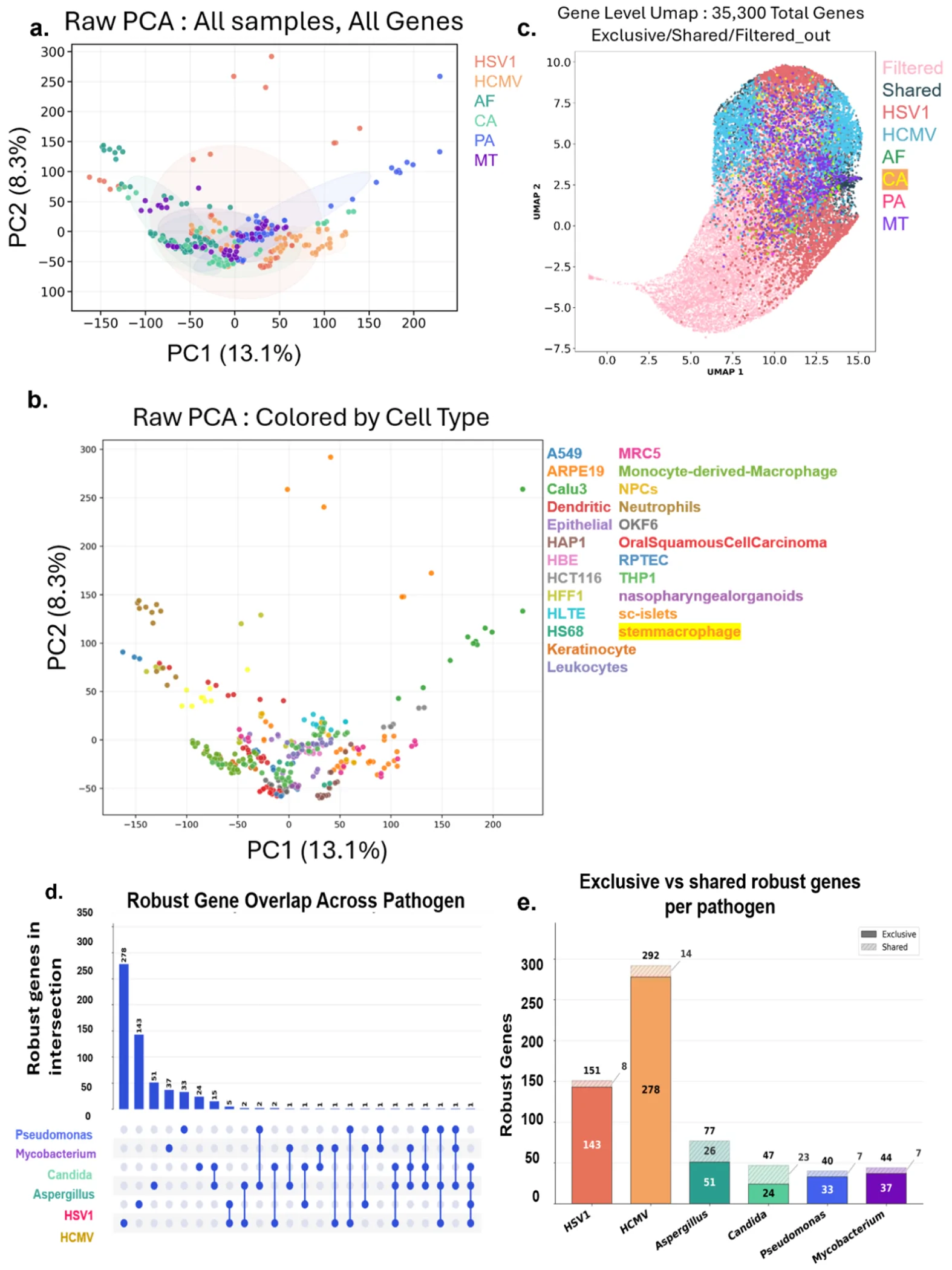
Samples representation and genes distribution. a. Raw PCA of all samples prior to filtering. PC1 and PC2 explain 13.1% and 8.3% of total variance, respectively. b. Raw PCA of all samples based on cell types. PC1 and PC2 together explain 21.4% of total variance. c. Gene-level UMAP of all 35,300 genes: Points show if a gene is exclusively associated with a single pathogen’s robust gene set, shared across two or more pathogens, or filtered out. d. UpSet plot of differentially expressed robust genes. e. Total robust genes per pathogen based on 651 gene pathogen entries across 605 unique genes, divided into genes exclusive to that pathogen (solid) and genes shared with at least one other pathogen (hatched). Total counts are shown above each bar.

**Figure 2:**
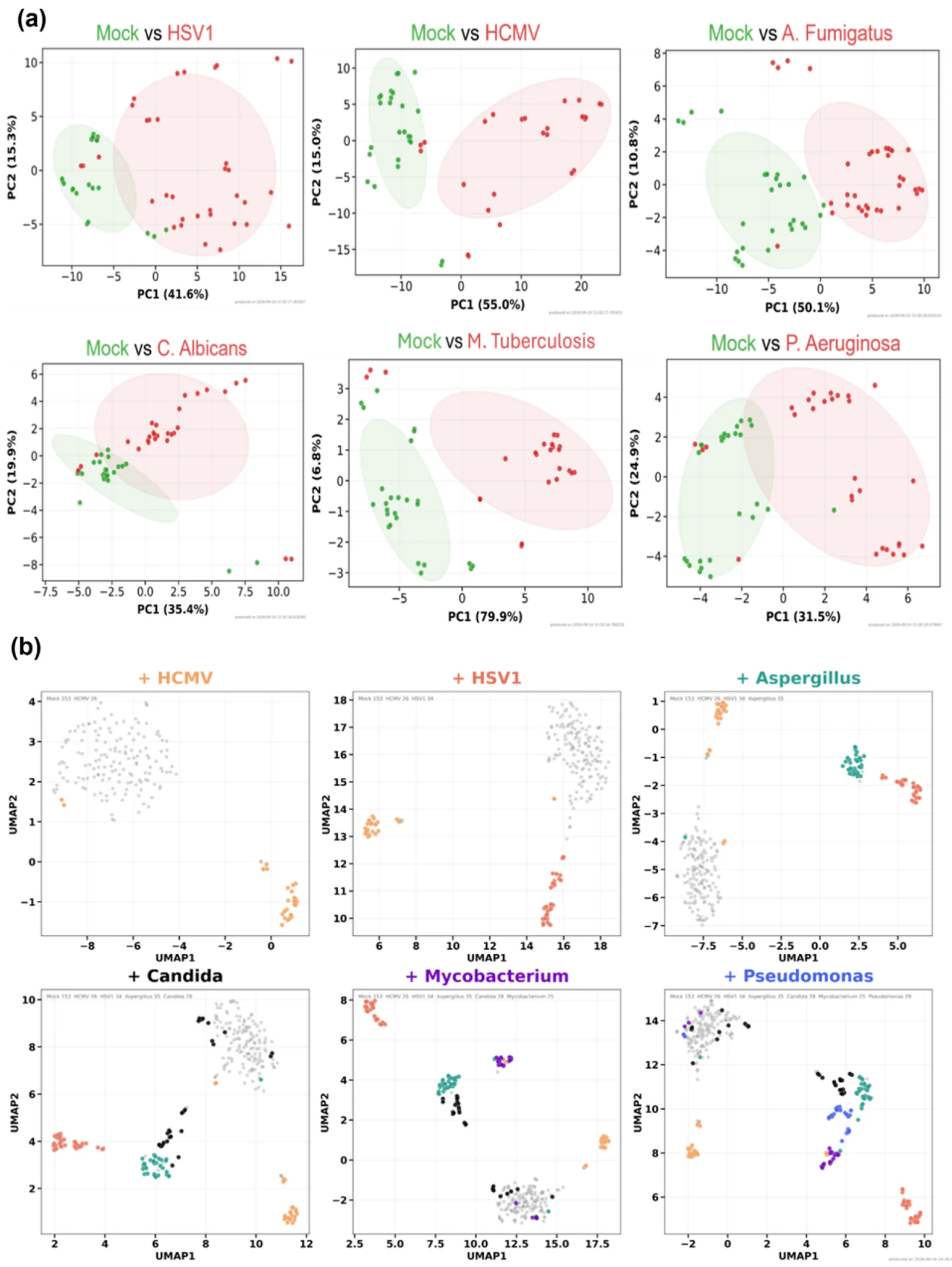
**a. PCA of infected versus mock samples restricted to pathogen-specific robust genes.** For each pathogen, samples were projected using only the robust genes identified for that pathogen via per-study differential expression (PyDESeq2), with genes required to show consistent differential expression across a minimum number of studies (indicated per panel). **(A)**Mock vs. HSV1 (151 robust genes, minimum 6 studies; PC1 = 41.6%, PC2 = 15.3%). **(B)**Mock vs. HCMV (292 robust genes, minimum 6 studies; PC1 = 55.0%, PC2 = 15.0%). **(C)**Mock vs. Aspergillus (77 robust genes, minimum 5 studies; PC1=50.1% and PC2=10.8%). **(D)** Mock vs. Candida (47 robust genes, minimum 4 studies; PC1 = 35.4% and PC2=19.9%). **(E)** Mock vs. M.Tuberculosis (44 robust genes, minimum 6 studies; PC1 = 79.9% and PC2= 6.8%). **(F)** Mock vs. Pseudomonas (40 robust genes, minimum 3 studies; PC1 = 31.5% and PC2=24.9%). Total 651 genes of all pathogens. Across pathogens, infected and mock samples show clear separation along PC1, with ellipses representing 1.5 standard deviations for each group. **b. Incremental UMAP visualization of pathogen separation using robust genes.**UMAP projections were generated by sequentially adding filtered samples from each pathogen to the pooled mock samples, illustrating how separation evolves as pathogens are introduced. **(Step 1)** Mock vs. HCMV. **(Step 2)**Mock, HCMV, and HSV-1 added. **(Step 3)** Mock, HCMV, HSV-1, and Aspergillus added. **(Step 4)** Mock, HCMV, HSV-1, Aspergillus, and Candida added. **(Step 5)** Mock, HCMV, HSV-1, Aspergillus, Candida and Mycobacterium added. **(Step 6)** Mock, HCMV, HSV-1, Aspergillus, Candida, Mycobacterium and Pseudomonas added. Each pathogen forms a visually distinct cluster relative to mock samples upon introduction, though clusters increasingly interleave with mock and one another as additional pathogens are added.

**Figure 3:**
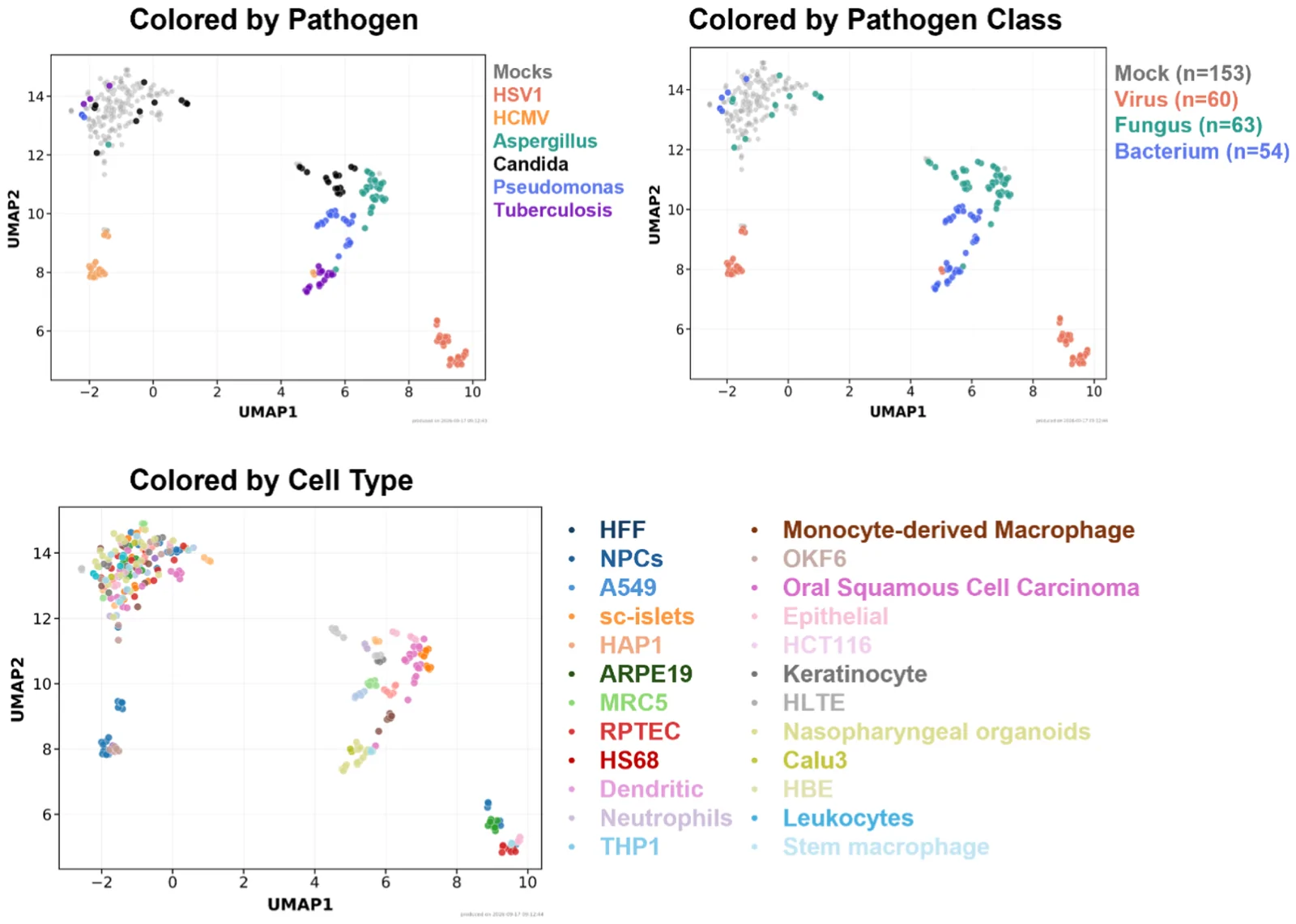
UMAP visualization of the combined robust-gene matrix (605 robust genes, 330 samples), colored by three grouping variables. **(A)**Colored by individual pathogen (HSV-1, n=34; HCMV, n=26; Aspergillus, n=35; Candida, n=28; Pseudomonas, n=29; Mycobacterium, n=25; Mock, n=153). **(B)**Colored by pathogen class (Virus, n=60; Fungus, n=63; Bacterium, n=54; Mock, n=153). **(C)** Colored by host cell type, included as a bias-check to visualize the distribution of cell types across the projection. Pathogen classes separate clearly from one another and this separation persists at the level of individual pathogens while several cell types appear across more than one pathogen cluster, indicating that the separation is not driven by cell-type identity alone.

**Figure 4:**
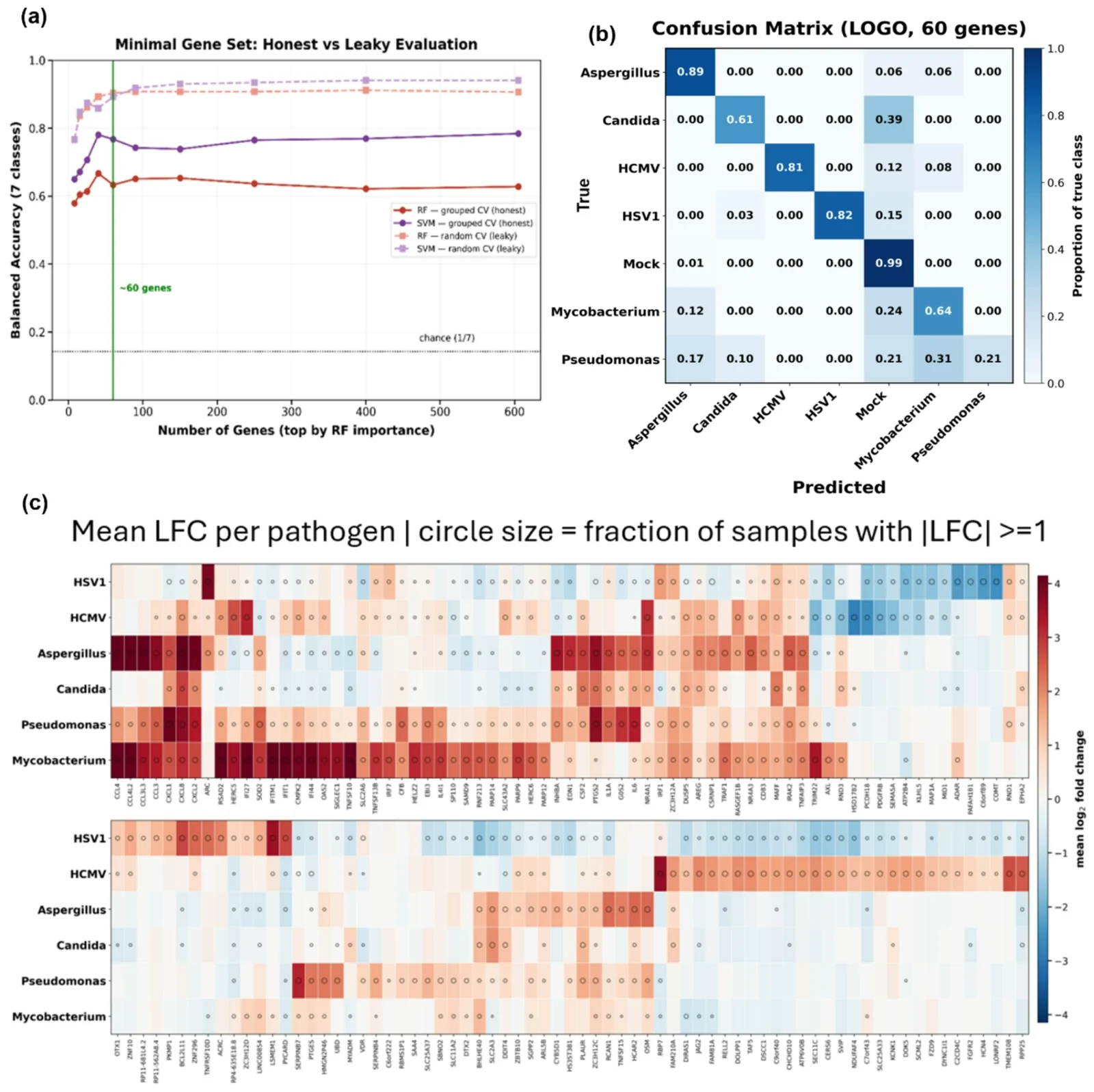
Results after applying the supervised machine learning classifier. **a.** Minimal-gene-set curve. Grouped CV (solid) stays flat for RF but rises gradually for SVM at larger gene counts, remaining far below the leaky random-split accuracy (dashed) throughout. **b.** Row-normalized confusion matrix (held-out studies, 60 genes). Mock, Aspergillus, HCMV and HSV-1 are recovered well. Candida and Mycobacterium Tuberculosis are moderately recovered; Pseudomonas Aeruginosa is frequently confused. **c.** Mean host response across pathogens for the 142 genes selected by random forest across cross-validation folds. Color indicates the mean log_2_ fold change relative to study-matched mock samples, averaged across all samples of each pathogen; red denotes upregulation and blue downregulation. Circle diameter indicates the proportion of that pathogen’s samples with |log_2_ fold change| ≥ 1, shown where this proportion is at least 0.15 (15% of samples). Genes are ordered by hierarchical clustering of their cross-pathogen response profiles.

**Figure 5:**
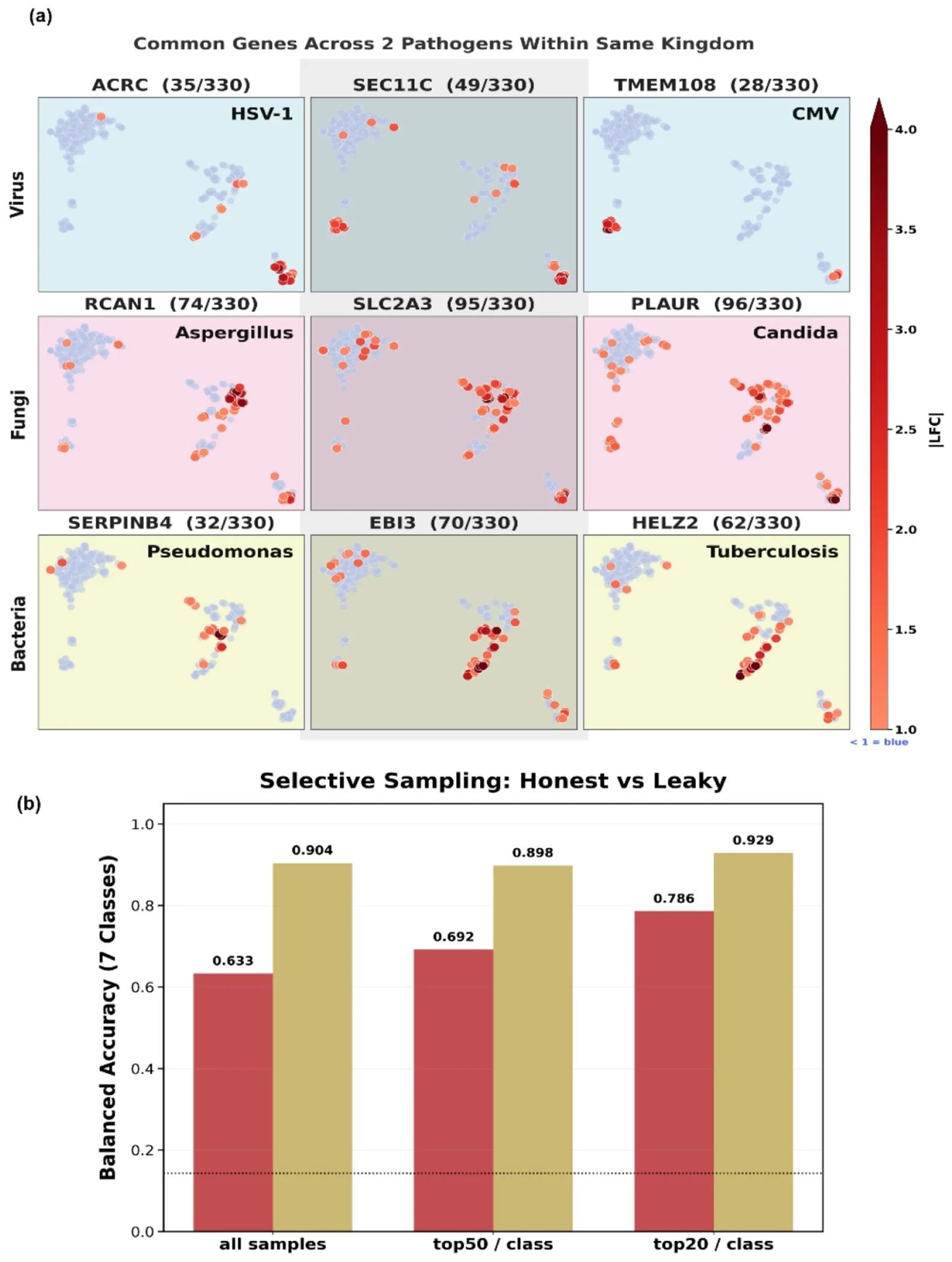
Gene-level response patterns and the effect of sample selection on classification accuracy. **a.** LFC>=1 relative to mockof representative pathogen-specific (left, right columns) and common (center column) marker genes projected onto the fixed sample-level UMAP, organized by pathogen class (rows: Virus, Fungus, Bacteria). Points are colored by intensity level (blue = low, red = high); fractions indicate samples with detectable expression (LFC) out of 330 total. Pathogen-specific genes show intensity localized to their corresponding cluster, while common genes are more broadly elevated across multiple clusters. **b.** Honest (grouped five-fold) versus leaky (random-split) balanced accuracy across sample-selection regimes. Random splits report 0.898–0.929 regardless of sample selection, whereas grouped cross-validation reports 0.633–0.786. Because subsetting reduced the Mock class and retained the strongest responders, differences between regimes reflect both class balance and sample response strength.

**Table 1.** Robust gene panels per pathogen vs. Mock comparison.

Comparison	Robust Genes	Minimum Studies	Total Studies
**Mock vs. HSV-1**	151	6	6
**Mock vs. HCMV**	292	6	7
**Mock vs. Aspergillus**	77	5	6
**Mock vs. Candida**	47	4	7
**Mock vs. M. tuberculosis**	44	6	7
**Mock vs. Pseudomonas**	40	3	5

**Table 2.** Cohort summary

Property	Value
Total samples	330
Infected	177
Mock (pooled)	153
Pathogens	6 (HSV-1, HCMV, AF, CA, PA, MT)
BioProjects (studies)	36
Host cell types	24
Robust genes (features)	605

**Table 3.** Seven-class balanced accuracy for full gene space, honest LOGO versus leaky random cross-validation (RF). Chance = 0.14.

# genes	Balanced accuracy - leave-one-group-out (honest)	Balanced accuracy - random split (leaky)
8	0.519	0.756
40	0.586	0.848
60	0.573	0.848
90	0.578	0.867
150	0.576	0.845
400	0.568	0.861
605	0.582	0.872

**Table 4.** Seven-class balanced accuracy for 605 robust panel genes, honest LOGO versus leaky random cross-validation (RF). Chance = 0.14.

# genes	Balanced accuracy - leave-one-group-out (honest)	Balanced accuracy - random split (leaky)
8	0.564	0.768
40	0.679	0.893
60	0.709	0.904
90	0.692	0.908
150	0.685	0.907
400	0.699	0.912
605	0.679	0.907

**Table 5.** Per-class recall under LOGO validation (60-gene Random Forest).

Class	n	Recall (LOGO, 60 genes)
Mock	153	0.99
HSV-1	34	0.82
Aspergillus	35	0.89
Mycobacterium	25	0.64
HCMV	26	0.81
Pseudomonas	29	0.21
Candida	28	0.61

**Table 6.** Seven-class balanced accuracy under selective sampling.

Subset	Balanced acc : LOGO (honest)	Balanced acc : random (leaky)
All infected	0.633	0.904
Top 50 / pathogen	0.692	0.898
Top 20 / pathogen	0.786	0.929

## Data Availability

1. studydesign.xlsx contains all the metadata for the samples. 2. The bulk RNASeq data was fetched and downloaded from NCBI Sequence Read Archive with Organism: Homo Sapiens, Layout : Paired or Single, Source : Transcriptomics and Selection : cDNA 3. All the samples were matched with the Ensembl Human Transcriptome. 4. The data folders consisting all 330 samples as xlsx files are available upon request as the folder size is large. 5. All the transcripts were converted to genes using the summation of isoforms and mapped using tx2gene data. 6. The main matrix of robust gene’s log fold change values is attached as combine_lfc_matrix.tsv. No new sequencing data were generated in this study. The RNA-seq datasets analysed are publicly available in the NCBI Sequence Read Archive (https://www.ncbi.nlm.nih.gov/sra) under BioProject accessions mentioned in the aforementioned excel file. Run-level (SRR) and BioSample accessions, with sample metadata for all 330 samples, are provided in Additional file 1 (studydesign.xlsx). Processed data generated during this study, including per-sample gene-level abundance matrices, the robust-gene log fold-change matrix (combine_lfc_matrix.tsv), per-pathogen robust gene lists and the 142 genes selected across cross-validation folds, are available in the Zenodo repository, https://doi.org/10.5281/zenodo.22905892. The analysis code is available at https://github.com/mariaplumber/Ocular-Pathogen-Classification and archived at the same Zenodo record.https://doi.org/10.5281/zenodo.22905892 (Plumber et al 2026)

## Abbreviations

HSV-1: herpes simplex virus 1
HCMV: human cytomegalovirus
AF: Aspergillus fumigatus
CA: Candida albicans
MT: Mycobacterium Tuberculosis
PA: Pseudomonas Aeruginosa
DEG: differentially expressed gene
LOGO: leave-one-group-out
mNGS: metagenomic next-generation sequencing
PCA: principal component analysis
PCR: polymerase chain reaction
RF: random forest
SVM: support vector machine
TPM: transcripts per million
UMAP: uniform manifold approximation and projection
